# Impact of high-speed shear homogenization pretreatment on structure, functional characteristics, and interfacial properties: A case of Rice Glutelin

**DOI:** 10.1016/j.fochx.2025.102219

**Published:** 2025-01-25

**Authors:** Zhuangpeng Wang, Zhangtao Chen, Lufan Tan, Jin Tu, Yong Sun, Yuanping Ye, Senwang Zhang, Leiyan Wu

**Affiliations:** a College of Food Science and Engineering*,* Jiangxi Agricultural University*,* Nanchang 330045*,* China; b Institute of Applied Chemistry*,* Jiangxi Academy of Sciences*,* Nanchang 330096*,* China; c State Key Laboratory of Food Science and Resources*,* Nanchang University*,* Nanchang 330047*,* China; d Jiangxi Riyuan Food Co.*,* Shangrao 334604*,* China

**Keywords:** High-speed shear homogenization, Rice glutelin, Structure, Functional Characteristics, Interfacial properties

## Abstract

In this study, rice glutelin (RG) was pretreated using high-speed shear homogenization (HSSH) to enhance its functional characteristics and interfacial properties through structural modification. Its structure was characterized using techniques such as SDS-PAGE, FT-IR, SEM, interface analyzer, dynamic and electrophoretic light scattering. The results indicated that HSSH preserved the primary structure of RG but significantly affected its secondary structure. It increased the surface hydrophobicity and conformational flexibility, enhanced electrostatic repulsion, reduced the particle size, and produced a loose and porous microstructure. These alterations resulted in variations in the functional and interfacial properties of RG. After HSSH treatment at 12,000 rpm for 2 min, RG exhibited optimal improvements in solubility (5.56 %), WHC (6.00 g/g) and OHC (2.20 g/g), EAI (10.19 m^2^/g) and ESI (341.98 min), as well as FC (16.20 %) and FS (64.21 %). However, excessive HSSH treatment induced the formation of aggregates, which is detrimental to the improvement of these properties.

## Highlights

• HSSH-treated RG became looser, more hydrophobic and more flexible.
• HSSH-treated RG has lower interfacial tension, faster *K*_d_, *K*_p_, *K*_*r*_, and stronger *E*_*d*_.
• The emulsifying and foaming capacities of HSSH-treated RG are enhanced.
• The emulsifying and foaming capacities of RG are closely tied to its structure and interfacial properties.

## 1 Introduction

Rice is a vital global food source known for its nutritional value and digestibility (Amagliani et al., 2017). Asia is the center of rice cultivation, consumption, and trade, with China and India producing approximately 51 % of global output (Singh et al., 2023). In China, more than 60 % of the population relies on rice as a dietary staple (Guo et al., 2023). Research indicates that rice not only provides essential calories that the human body needs every day, but also offers a variety of nutrients, including proteins, fats, carbohydrates, fiber, minerals, and vitamins (Hashimoto et al., 2022). As the second largest nutrient component in rice after starch, rice protein is particularly beneficial for functional foods owing to its low allergenicity and effectiveness, which are comparable to those of bovine casein (Zhao et al., 2020). Additionally, hydrolyzed rice protein has various biological functions such as antioxidant properties and benefits in anti-hypertensive, anti-obesity, and anti-cancer (Ren et al., 2021). However, rice protein is a by-product of starch syrup, lactic acid, monosodium glutamate, and alcohol production and can often be used as animal feed or for non-food purposes, leading to ecological pollution and wastage of protein resources (Zhao et al., 2020). Moreover, composed primarily of albumin, globulin, gliadin, and glutelin, with glutelin making up approximately 80 %, the properties of rice protein are largely determined by glutelin (Yang et al., 2023). Nevertheless, the inherent structural rigidity of RG limits its molecular flexibility and reduces its solubility and surface activity under neutral conditions (Zhao et al., 2020), hindering its functional application in food products. Therefore, novel approaches are required to modify RG structure and enhance its functional and interfacial properties for more effective use as a functional food ingredient.

High-speed shear homogenization (HSSH) is a safe and environmentally friendly technique used in energy-intensive processes such as homogenization, dispersion, emulsification, grinding, dissolution, and cell disruption across agriculture, food production, and chemical reactions (Zhang et al., 2012). It breaks larger particles into smaller ones through the shear force generated by the high-speed movement of the rotor as well as the effects of laminar flow, turbulence, and cavitation to achieve rapid and uniform mixing of the particles (Zhou et al., 2019). Recent studies have demonstrated that HSSH can enhance protein functional and interfacial properties such as solubility and emulsification. For instance, it improves the emulsifying ability and stability of myofibrillar proteins by exposing hydrophobic domains on the surface of smaller particle (Zhou et al., 2019). It can also enhance the solubility of walnut protein by reducing its particle size and improving its interaction with water molecules (Kong et al., 2019). Moreover, it reduces soy protein isolate aggregation and significantly increases solubility via the application of intense shear forces (Zou et al., 2019). Although proteins such as myofibrillar and walnut proteins can exhibit positive changes with HSSH treatment, RG may have distinct characteristics. The effects of HSSH on RG properties remain unknown, whereas HSSH pretreatment might modify the functional and interfacial properties of RG, broadening its application in commercial food products.

Based on previous findings, further investigation of the impact of HSSH on RG functional and interfacial properties is necessary, as similar improvements have been observed in other proteins. Understanding how HSSH alters RG functional and interfacial characteristics may provide new opportunities for its inclusion in various food applications. This study can enable food manufacturers and researchers to develop strategies to utilize this underutilized protein more effectively. Overall, exploring HSSH as a pretreatment technique for RG can provide valuable insights into enhancing its functionality and expanding its potential applications in multiphase systems such as cakes or emulsions.

## 2 Materials and methods

### 2.1 Material

Rice protein samples were obtained from Hengding Food Co., Ltd. (Fengcheng, China). Petroleum ether, sodium chloride, and ethanol were supplied by Xilong Science Co., Ltd. (Shantou, China). Precast protein gel was provided by Boyi Biotechnology Co., Ltd. (Changzhou, China). Protein loading buffer (5 ×), pre-stained protein markers, and Coomassie Brilliant Blue fast staining solution were obtained from Servicebio Biotechnology Co., Ltd. (Wuhan, China). Trypsin and trichloroacetic acid were supplied by Macklin Biochemical Technology Co., Ltd. (Shanghai, China). A 0.05 mol/L (pH 8.0) Tris-HCl buffer was sourced from Yuanye Biotech Co., Ltd. (Shanghai, China), and potassium bromide was provided by Aladdin Bio-Chem Technology Co., Ltd. (Shanghai, China). Sodium dodecyl sulfate and BCA Protein Assay Kit were supplied by Solarbio Technology Corporation Limited (Beijing, China). The Florisil adsorbent was sourced from Sigma-Aldrich Corporation (USA), and soybean oil was obtained from Bangge Grain & Oil Company Limited (Nanjing, China). All reagents used, unless otherwise specified, were of analytical grade.

### 2.2 Determination of basic indicators of rice protein

The analysis of crude protein, crude fat, moisture, and ash contents of rice was based on GB5009.5–2016, GB5009.6–2016, GB5009.3–2016, and GB5009.4–2016, respectively. The analysis results are presented in Table S1.

### 2.3 Preparation of RG

The extraction process of RG followed the methods outlined in previous studies (Zhao et al., 2020). After extraction, RG was lyophilized and preserved at 4 °C for future applications. The Kjeldahl method demonstrated that RG content was 86.9 % on a dry weight basis (N × 5.95).

### 2.4 HSSH pretreatment of samples

Following minor modifications from previous literature (Cui et al., 2022), the RG solution (10 mg/mL) was prepared by dissolving RG powder in ultrapure water (pH 6.56) and stirring on a magnetic stirrer (IKA Group RW2, Staufen, Germany) at ambient temperature for 1 h prior to shear homogenization. The solution (60 mL) was placed in a 100 mL glass flask and subjected to shear at various speeds, including 11,000, 12,000, 13,000, 14,000, and 15,000 rpm, for 2 min. All samples were cooled in an ice bath to prevent heat generation during homogenization, thus affecting the results. As a control, RG was not subjected to high-speed shear homogenization. The samples were freeze-dried and stored at 4 °C until further utilization.

### 2.5 Determination of protein structure

#### 2.5.1 Gel electrophoresis analysis (SDS-PAGE)

SDS-PAGE was conducted using electrophoresis equipment (DYY-6D, Liuyi Biotechnology Co., Ltd., Beijing, China). The sample was mixed with 5× loading buffer and heated in a boiling water bath for 5 min. A total of 10 μL of the sample prepared with a 4 % stacking gel and a 12 % separating gel was run at 160 V for 45 min. After electrophoresis, the gels were rinsed three times with ultrapure water, stained with Coomassie Brilliant Blue Ultrafast Staining Solution for 6 h, and rinsed again three times with ultrapure water. The gels were stored overnight until the appearance of clear bands.

#### 2.5.2 Average particle size and zeta-potential

The particle size and zeta potential of the samples were measured by dynamic light scattering and electrophoretic light scattering, respectively, following a slightly modified version of an established method (Zhang et al., 2021). A Zetasizer Nano ZS90 (Malvern Instruments, UK) was used for the measurements. The samples were diluted to a concentration of 10 mg/mL in ultrapure water under ambient conditions (25 °C), and each measurement was performed three times.

#### 2.5.3 Fourier transform infrared analysis (FT-IR)

Infrared spectra (Nigolis Instruments, Madison, USA) of RG were recorded using the potassium bromide pellet method (Wang et al., 2016). The experimental data were processed using OMNIC8 (Nigolis Instruments, Madison, USA) and PeakFit v4.12 (SeaSolve Software Inc., USA).

#### 2.5.4 Conformational flexibility

Conformational flexibility was following the method outlined in reference (Li et al., 2019) with minor modifications, trypsin was diluted to the concentration of 10 mg/mL using a 0.05 mol/L Tris-HCl buffer at pH 8.0 to prepare the enzyme solution. Subsequently, 250 μL of this enzyme solution was combined with 4 mL of a protein solution (diluted to 10 mg/mL in ultrapure water) and incubated in a water bath at 38 °C for 5 min. The reaction was terminated by adding 4 mL of 5 % (*w*/*v*) trichloroacetic acid solution. After centrifugation at 8000 rpm for 15 min, the supernatant was analyzed using a UV spectrophotometer (UV-5200PC, Shanghai Yuanxi Instrument Co., Ltd., China) at an absorbance wavelength of 280 nm to assess flexibility based on absorbance *A*_0_.

#### 2.5.5 Surface hydrophobicity

Surface hydrophobicity was determined following the method outlined in a previous study (Zhao et al., 2020) with slight modifications. The three-phase contact angle of RG was measured before and after pretreatment using an interface analyzer (OSA100, Ningbo New Frontier Scientific Instrument Co., Ltd., China).

#### 2.5.6 Scanning electron microscope (SEM)

The morphology of freeze-dried RG samples was examined using a scanning electron microscope (Carl Zeiss EVO18, Zeiss Group, Oberkochen, Germany). The protein samples were coated with gold under vacuum and affixed to a double-sided conductive adhesive. Surface microstructure variations in RG before and after pretreatment were observed at an accelerating voltage of 3 kV.

### 2.6 Evaluation of the functional and interfacial characteristics of RG

#### 2.6.1 Assessment of the solubility

The solubility assay followed minor adaptations of the method described in a previous study (Wang et al., 2022). The samples were suspended in ultrapure water (pH 6.56) and gently stirred using a magnetic stirrer at room temperature for 1 h to form an RG solution (10 mg/mL). The dispersion was then centrifuged at 8000 rpm for 15 min. The protein content of the supernatant was measured using a BCA protein assay kit to evaluate the solubility variations of RG under different treatment conditions.

(1) $$\text{Solubility}\left(\%\right)=\frac{\text{protein content in supernatant}}{\text{total protein content before centrifugation}}\times 100\%\#$$

#### 2.6.2 Evaluation of the water holding capacity and oil holding capacity

The water holding capacity (WHC) and oil holding capacity (OHC) were determined following the method described in a previous study (Ding et al., 2021) with slight modifications. Lyophilized RG powder (0.1 g) was mixed thoroughly with 1 mL of ultrapure water or soybean oil in a 1.5 mL centrifuge tube and left to sit for 30 min at room temperature. The mixture was then centrifuged at 8000 rpm for 5 min to separate the supernatant. The calculation formula involved the following: *M*_0_ represents the weight of the powdered sample (g), *M*_1_ the total weight (g) of the centrifuge tube and sample, *M*_2_ the total weight (g) of the centrifuge tube with the sediment containing water, and *M*_3_ the total weight (g) of the centrifuge tube with the sediment containing oil.

(2) $$\begin{array}{c} \mathrm{WHC}\left(\frac{g}{g}\right)=\frac{M_{2}-M_{1}}{M_{0}}\# \end{array}$$

(3) $$\begin{array}{c} \mathrm{OHC}\left(\frac{g}{g}\right)=\frac{M_{3}-M_{1}}{M_{0}}\# \end{array}$$

#### 2.6.3 Oil purification

Purified edible soybean oil was used to evaluate the oil-water interfacial characteristics of the proteins. The purification process involved the addition of 8 g of Florisil molecular sieve to 200 mL of soybean oil followed by magnetic stirring and settling. The mixture was then centrifuged at 8000 rpm for 15 min to remove the molecular sieve. This procedure was repeated with fresh molecular sieves until no further changes in the interfacial tension between soybean oil and ultrapure water were observed.

#### 2.6.4 Determination of interfacial tension

The changes in the interfacial tension at the air-water or oil-water interfaces in different samples were dynamically assessed using an interface analyzer (OSA100, Ningbo New Boundary Scientific Instrument Co., Ltd., China). A 10 μL droplet of RG solution was created at the injection needle, which was inserted into a colorless glass reaction vessel. For the air-water interface evaluation, ultrapure water was introduced into the reaction cup and covered with an aluminum foil to prevent evaporation. For the oil-water interface evaluation, purified soybean oil was added to the vessel. The droplet shapes were continuously recorded for 2000 s at a rate of one data point per second using a camera. The dynamic interfacial tension values for each sample were then calculated based on droplet shape analysis using the Laplace-Young equation.

#### 2.6.5 Interfacial adsorption kinetics

The initial stage of the adsorption mechanism is typically controlled by the diffusion rate (*K*_d_, mN/m^−1^·s^-0.5^) of proteins, which can be quantified using the Ward–Tordai formula.

(4) $$\begin{array}{c} \pi =2\cdot C_{0}\cdot K\cdot T\cdot {\left(\frac{\mathrm{Dt}}{3.14}\right)}^{\frac{1}{2}}\# \end{array}$$

where *C*_0_ represents the protein concentration (g/mL) in the bulk phase, *K* is the Boltzmann constant, *T* is the absolute temperature (°C), *D* is the diffusion coefficient of the protein, and *t* is the adsorption time (s). This equation allows investigation of the kinetics of protein molecule permeation and rearrangement at the interface.

(5) $$\begin{array}{c} \ln\left[\frac{\left(\pi_{2000}-\pi_{t}\right)}{\left(\pi_{2000}-\pi_{0}\right)}\right]=-\mathrm{kit}\# \end{array}$$

where *π*_2000_, *π*_0_, and *π*_*t*_ denote the interfacial pressures at 2000 s, 0 s, and any specific second, respectively, and *K*_*i*_ represents the first-order rate constant. The graph derived from this equation illustrates two distinct linear regions: the initial slope represents the rate constant (*K*_p_, s^−1^) for the permeation of protein molecules at the interface and the subsequent slope reflects the rearrangement rate (*K*_*r*_, s^−1^) of these molecules at the interface.

#### 2.6.6 Determination of interfacial expansion rheology

This method was adapted from a previous study (Xiong et al., 2018). The experiment conducted at 25 °C for 2000 s involved sinusoidal compression and expansion of the interface by adjusting the droplet volume within a linear range corresponding to 10 % deformation amplitude. The oscillation frequency was maintained at 0.1 Hz, with an initial RG solution droplet volume of 8 μL. After a 60-s stabilization period, the experimental process began.

#### 2.6.7 Determination of emulsion and foam microstructure

The morphologies of the fabricated emulsions and foams were examined using an optical microscope (Chongqing Aopu Optoelectronics Technology Co., Ltd., China). Specifically, 100 μL of each sample was placed on a glass slide, covered with a cover slip, and observed under a microscope with a 40× objective lens.

#### 2.6.8 Assessment of emulsifying activity and emulsion stability

The emulsifying activity (EAI) and emulsion stability (ESI) were evaluated as described in a previous study (Hu & Li, 2022) was followed with minor adjustments. A protein solution (10 mg/mL) was mixed with soybean oil at a 4:1 ratio and homogenized for 2 min at 10,000 rpm using a high-speed homogenizer (IKA ULTRA-TURRAX T25 Staufen, Germany) to form the emulsion. Subsequently, 100 μL of the emulsion was removed from the beaker and added to 5 mL of 0.1 % sodium dodecyl sulfate (SDS) solution. The absorbance of the mixed solution was measured at 500 nm using a UV spectrophotometer (UV-5200PC, Shanghai Yuanxi Instrument Co., Ltd., China). The EAI and ESI values were calculated using an equation based on the dilution factor (*N* = 50), protein concentration (*C*, g/mL), oil phase fraction (θ = 0.2), and absorbance readings taken at 0 min (*A*_0_) and 10 min (*A*_10_).

(6) $$\begin{array}{c} \mathrm{EAI}\left(\frac{m^{2}}{g}\right)=\frac{2\times 2.303\times A_{0}\times N}{C\times \theta \times 10000}\# \end{array}$$

(7) $$\begin{array}{c} \mathrm{ESI}\left(\min\right)=\frac{A_{0}}{A_{0}-A_{10}}\times 10\# \end{array}$$

#### 2.6.9 Evaluation of foaming capacity and foam stability

The foaming capacity (FC) and foam stability (FS) were evaluated as described in a previous study (Lan et al., 2020) was followed with minor adjustments. RG solution (5 mL, 10 mg/mL) was homogenized at 10,000 rpm for 2 min using a high-speed homogenizer. The foam volume for each sample was recorded at 2 and 30 min after homogenization. The FC and FS values were then calculated using the following parameters: 5 represents the initial volume of the protein solution before homogenization, *V*_2_ is the foam volume after 2 min, and *V*_30_ is the foam volume after 30 min of stabilization under ambient conditions.

(8) $$\begin{array}{c} \mathrm{FC}\left(\%\right)=\frac{V2}{5}\times 100\# \end{array}$$

(9) $$\begin{array}{c} \mathrm{FS}\left(\%\right)=\frac{V30}{\begin{array}{c} \text{V2} \end{array}}\times 100\# \end{array}$$

### 2.7 Statistical analysis

Unless otherwise specified, all tests were conducted in triplicate. Significant differences (*P* < 0.05) were analyzed using analysis of variance (ANOVA) in SPSS 22.0 (SPSS Inc., Chicago, IL, USA). Additionally, the Duncan test was used for multiple comparisons. Mapping and Pearson correlation analyses were performed using Origin 2022 (OriginLab Corporation, Northampton, USA).

## 3 Results and discussion

### 3.1 Particle size, zeta potential, and SDS-PAGE

**Fig. 1:**
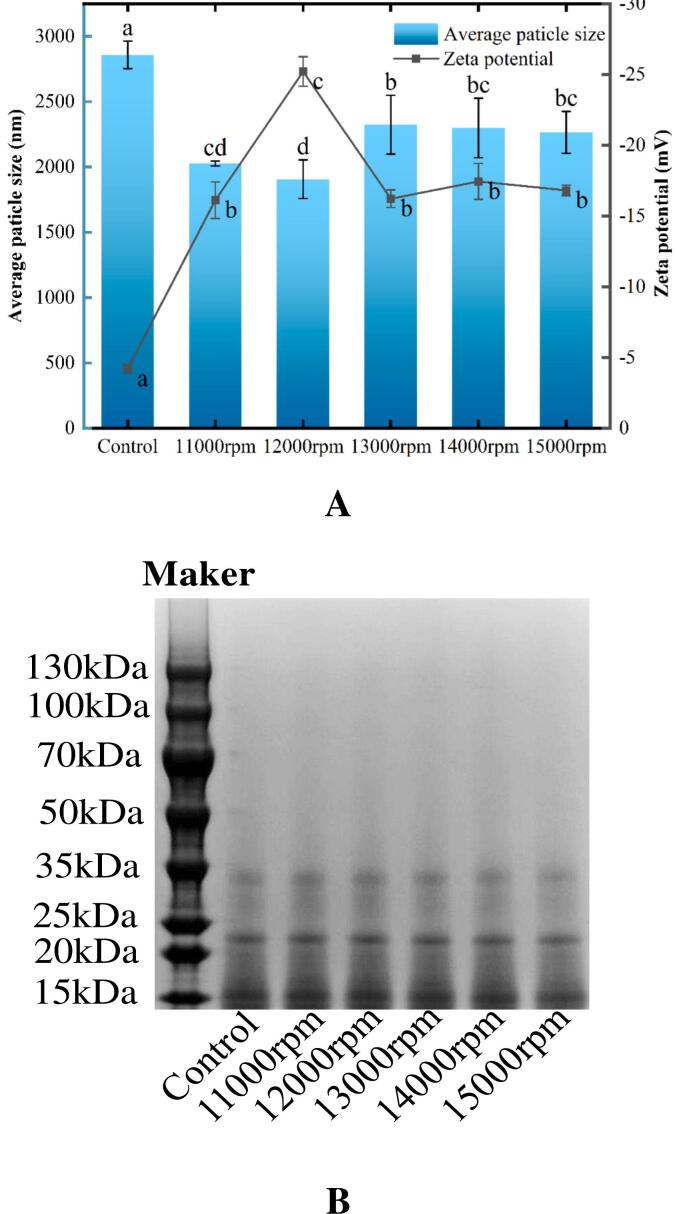
Impact of shear pretreatment at various shear speeds on the average particle size, zeta potential (A), and molecular weight (B) of RG.

The effects of the shear speed on the average particle size, zeta potential, and primary structure of RG are shown in Fig. 1. As illustrated in Fig. 1A, the sheared RG exhibited a smaller particle size and higher zeta potential, with a minimum particle size of 1906.58 nm and a maximum zeta potential of −25.21 mV at a shear rate of 12,000 rpm. The reduction in particle size was likely due to protein collisions under shear forces, which re-dissociated intermolecular aggregates and further increased electrostatic repulsion between protein molecules. This phenomenon can also be observed in dehulled walnut proteins, where shearing breaks down larger particles into smaller ones, increasing the surface area and charging sites (Kong et al., 2019). Interestingly, as the shear rate further increased (from 13,000 rpm to 15,000 rpm), the average particle size of RG gradually increased, while the zeta potential gradually decreased. This might be because the faster shear speed induced the re-aggregation of the disaggregated RG molecules (Kong et al., 2019; Zhou et al., 2019).

Although the shear force can alter the particle size and zeta-potential of RG, it was not sufficient to completely degrade the primary structure, as confirmed by SDS-PAGE (Fig. 1B). Both untreated and treated RG displayed three bands at molecular weights of 16, 23, and 34 kDa, indicating no significant change in the molecular weight. Unlike enzymatic hydrolysis or pyrolysis, which decomposes the primary structure of proteins (Ashraf et al., 2024; Li et al., 2021), physical treatments, such as high pressure, shearing, or ultrasound caused protein molecular collisions without altering the primary structure, resulting in less pronounced changes in the primary structure (Ding et al., 2021; Wu et al., 2022; Zhou et al., 2019).

### 3.2 FT-IR and conformational flexibility

**Fig. 2:**
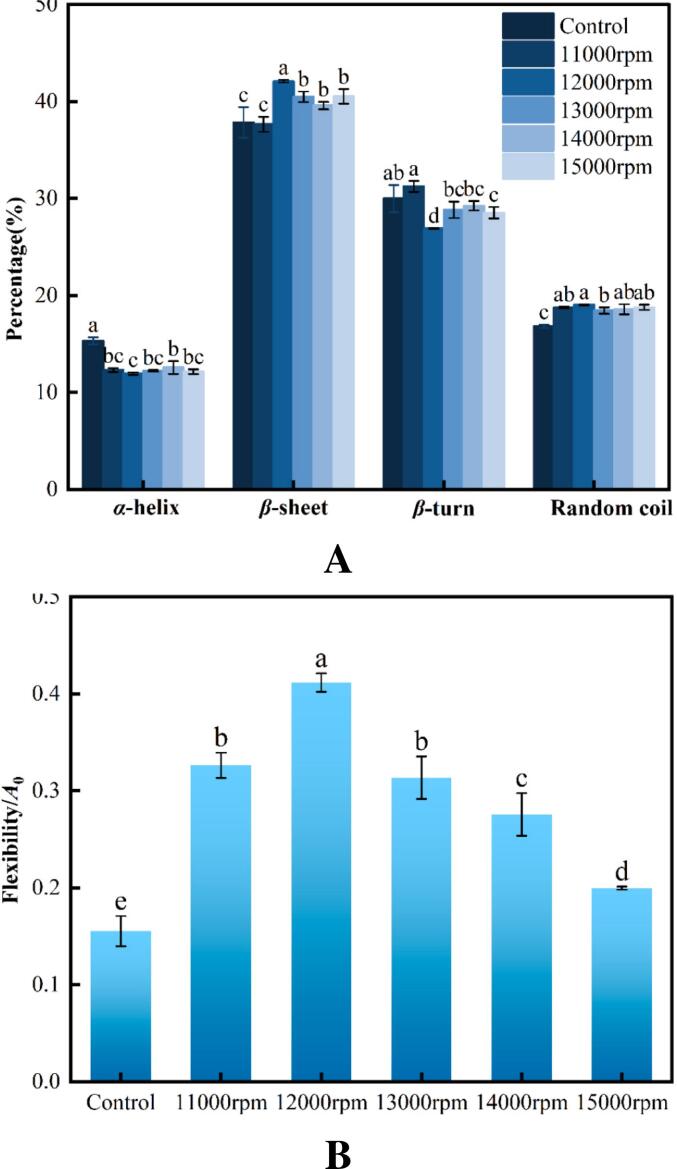
Impact of shear pretreatment at various shear speeds on the secondary structure (A) and conformational flexibility (B) of RG.

*FT-IR* analysis revealed a relationship between protein structure and function. As shown in Fig. 2A, compared with the control, shear treatment increased the content of RG *β*-sheet (except at 11000 rpm) and random coil structures while reducing the *α*-helix and *β*-turn structures. An increase in *β*-sheet content exposes hydrophobic regions (Zhang et al., 2014), indicating the unfolding of protein molecules (Taha et al., 2018), which can enhance conformational flexibility. Fig. 2B demonstrates that shear treatment significantly increased RG conformational flexibility (*P* < 0.05). However, this increase in conformational flexibility was not directly correlated with a higher shear speed. RG exhibited the greatest conformational flexibility at 12,000 rpm, which corresponded to the changes observed in the *β*-sheet conformation of the protein secondary structures. This phenomenon may be attributed to the strong laminar, turbulent, and cavitation effects generated by shear treatment, which disrupt hydrogen bonds and hydrophobic interactions within RG molecules, causing the decomposition of rigid protein structures and increased conformational flexibility (Yan et al., 2021a). These findings align with the behavior of ultrasound-treated soy proteins, where sonication unfolded spherical proteins during emulsification by exposing hydrophobic regions on protein surfaces, enhancing conformational flexibility, and promoting the formation of a stronger protein membrane at interfaces (Taha et al., 2018). Additionally, the solubility and structure of the polypeptide backbone also play a role in influence protein conformational flexibility (Zhu et al., 2020).

### 3.3 Surface hydrophobicity and scanning electron microscopy (SEM)

**Fig. 3:**
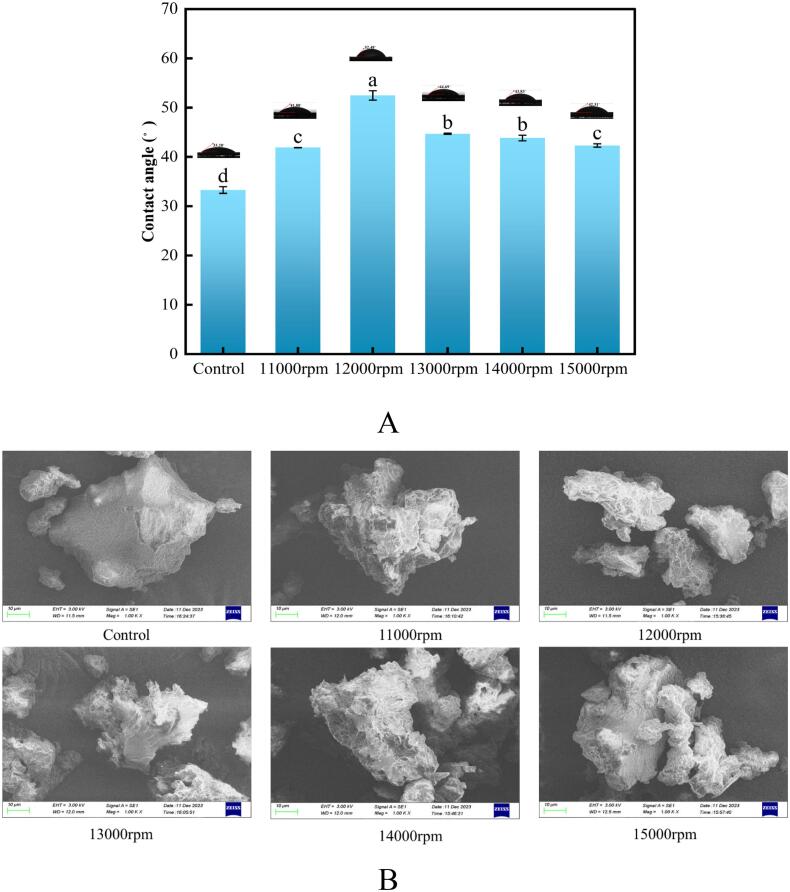
Impact of shear pretreatment at various shear speeds on the surface hydrophobicity (A) and scanning electron micrographs (B) of RG.

The three-phase contact angle reflects the surface hydrophobicity of proteins, which influences their solubility as well as their emulsifying and foaming properties (Alavi et al., 2019; Yan et al., 2021b). As shown in Fig. 3A, the air-liquid-solid three-phase contact angles of RG increased with increasing shear speed, indicating enhanced hydrophobicity (Zhao et al., 2020). This enhancement was likely due to the unfolding of protein molecules during high-speed shearing, which exposed more hydrophobic groups (Wang et al., 2021). However, surface hydrophobicity peaked at 12,000 rpm (52.48°) and gradually decreased to 42.31° at 15,000 rpm. These findings were consistent with those observed in mussel myofibrillar proteins treated with high-pressure homogenization. As the homogenization pressure increased from 0 to 80 MPa, the surface hydrophobicity gradually increased, reaching a peak at 80 MPa. However, it decreased at 100 MPa. It has been hypothesized that protein aggregation may be responsible for the decrease in surface hydrophobicity under these treatment conditions (Yu et al., 2018). Hence, this may explain the reduced surface hydrophobicity observed in RG after shearing treatment. The SEM images of RG support this conclusion. As shown in Fig. 3B, the sheared RG exhibited more  pores and cracks, further indicating that the protein unfolded during the shearing process, thereby exposing more  hydrophobic moieties and increasing hydrophobicity. This may be attributed to the exposure of extra charged sites on RG after shearing, which enhances the electrostatic repulsion force between RG molecules (Fig. 1A), resulting in the formation of more pores and cracks in sheared RG. These findings are consistent with those observed for shear-treated dehulled walnut proteins, where increased electrostatic repulsion prevents protein aggregation (Kong et al., 2019).

### 3.4 Solubility and water and oil holding capacity

Solubility is a key indicator of protein aggregation and denaturation, and significantly affects functions such as emulsification and foaming. As shown in Table 1, the solubility of RG increased with increasing shear speed, reaching a maximum of 5.56 % at 12,000 rpm. This was attributed to the dissociation of protein aggregates owing to the exposure of surface charges during shearing, which reduced the particle size and enhanced solubility (Li et al., 2023). This effect is also influenced by the protein surface microstructure (Zhang et al., 2020). However, solubility decreased when the shear speed exceeded 12,000 rpm. Comparable results were observed for sonicated sunflower protein isolates, where over-sonication led to protein denaturation and aggregation (Malik et al., 2017), which could explain the decrease in RG solubility. Interestingly, the W/OHC trend of RG was consistent with that of solubility, with sufficient W/OHC being crucial for food production processes such as sausages, batters, and baked goods (Ghanghas et al., 2020). As shown in Table 1, the sheared RG exhibited higher W/OHC values than untreated proteins, with the maximum WHC (6.00 g/g) and OHC (2.20 g/g) achieved at a shear rate of 12,000 rpm. These findings are consistent with the effects of sonication on scallop mantle proteins, where improved interactions between the exposed hydrophilic and hydrophobic groups and water/oil molecules were observed (Ding et al., 2021), potentially explaining the enhanced W/OHC after shear treatment. Nevertheless, as the shear rate further increased (from 13,000 rpm to 15,000 rpm), the W/OHC gradually decrease. This may be due to the reformation of RG aggregates induced by the higher shear rate, which causes the exposed hydrophilic and hydrophobic groups to be re-hidden. This change instead weakens the interactions between the hydrophilic and hydrophobic groups and water/oil molecules, resulting in the deterioration of the W/OHC.

**Table 1:** Impact of shear pretreatment at various shear speeds on the solubility and water/oil holding capacity (W/OHC) of RG.

Samples	Solubility (%)	WHC (g/g)	OHC (g/g)
Control	4.33 ± 0.04^c^	4.00 ± 0.00^c^	1.20 ± 0.10^c^
11,000 rpm	5.36 ± 0.16^ab^	5.37 ± 0.55^b^	1.63 ± 0.21^b^
12,000 rpm	5.56 ± 0.10^a^	6.00 ± 0.00^a^	2.20 ± 0.17^a^
13,000 rpm	5.32 ± 0.11^ab^	5.67 ± 0.58^ab^	1.77 ± 0.12^b^
14,000 rpm	5.31 ± 0.03^ab^	5.27 ± 0.35^b^	1.67 ± 0.06^b^
15,000 rpm	5.07 ± 0.48^b^	5.10 ± 0.00^b^	1.60 ± 0.10^b^

### 3.5 Interfacial tension

**Fig. 4:**
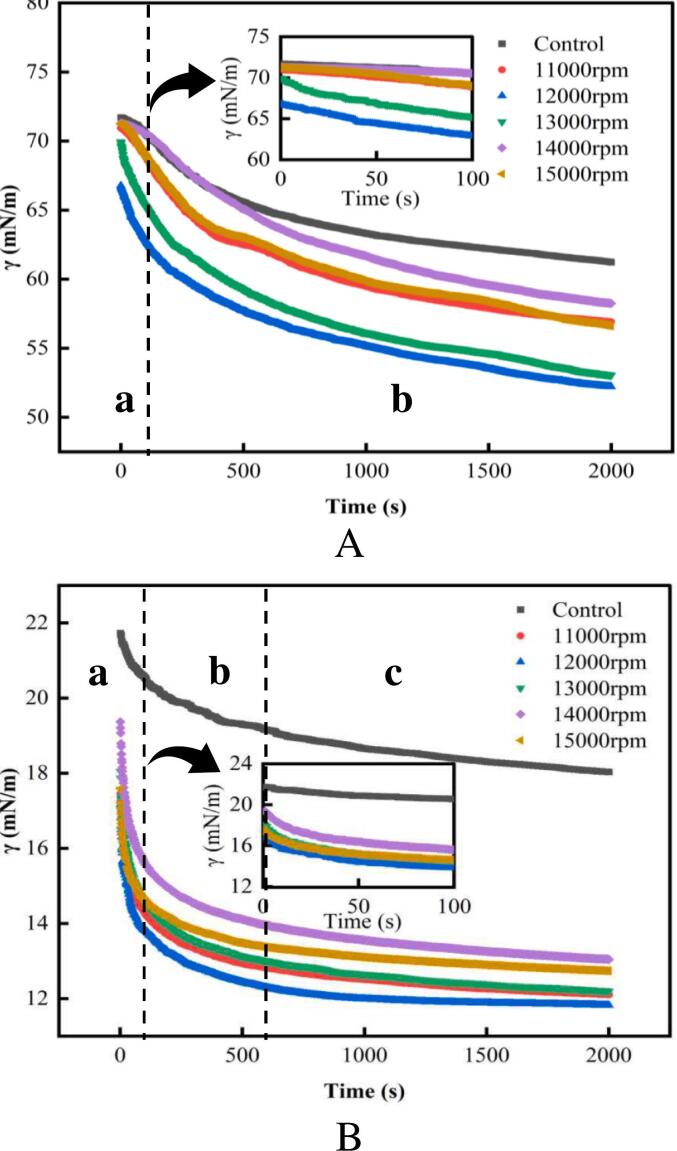
Dynamic interfacial tension curves of control and HSSH-treated RG at air-water (A) and oil-water (B) interfaces.

Interfacial tension provides dynamic information about the adsorption of molecules at air-water or oil-water interfaces, revealing their adsorption capacities (Sun et al., 2024). This study investigated the impact of shear treatment at different speeds on the interfacial adsorption behavior of RG by analyzing changes in interfacial tension. As shown in Fig. 4, the interfacial tension of RG decreased over time and eventually tended toward equilibrium at both the air-water or oil-water interfaces. This change could be due to increased electrostatic repulsion and steric hindrance as protein molecules adsorb at the interface, hindering further adsorption (Tao, Cai, Wang, Chen, Zhou, Yang, & Xu, 2024). Compared with the control, shear treatment significantly reduced the RG interfacial tension, suggesting that shear treatment could enhance the RG adsorption capacity at the interface. Further studies revealed that under the same shear time, increasing the shear speed resulted in a faster reduction in RG interfacial tension compared to the control, indicating that a higher speed accelerated the molecular adsorption at the interface. Specifically, RG exhibited the strongest adsorption capacity at the two-phase interface when the shear speed was 12,000 rpm, causing the interfacial tension to decrease to its lowest level, from 66.77 mN/m to 52.21 mN/m at the air-water interface and from 17.16 mN/m to 11.82 mN/m at the oil-water interface. This reduction could be attributed to the smaller particle size (Fig. 1A), higher solubility (Table 1), and greater surface hydrophobicity (Fig. 3A) of RG induced by a shear rate of 12,000 rpm. These changes act synergistically to enhance the adsorption capacity at the interface, ultimately leading to a rapid decrease in the interfacial tension. Previous studies have indicated that the reduction in interfacial tension is influenced by solubility, particle size, and surface hydrophobicity (Yang et al., 2018; Zhang, Liu, et al., 2025) rather than by a single factor. Interestingly, when the shear rate exceeded 12,000 rpm, the adsorption capacity of RG at the two-phase interface weakened, resulting in a further increase in the interfacial tension at the air-water or oil-water interface. This might be because an excessive shear rate causes the reformation of RG aggregates, which in turn leads to an increase in the particle size of protein molecules (Fig. 1A), decreased solubility (Table 1), and reduced surface hydrophobicity (Fig. 3A), thereby hindering the adsorption of RG molecules at the interface. Therefore, an appropriate shear treatment could enhance the RG adsorption capacity at the air-water or oil-water interfaces by altering their structural characteristics, leading to a more effective reduction in interfacial tension.

Protein adsorption at interfaces can be driven by the forces between two immiscible molecules resulting from the energy difference between the molecules at the fluid interface and those in the bulk phase. This compels the interface-active molecules to adsorb at the interface, reducing the Gibbs free energy and increasing stability (Berry et al., 2015; Li et al., 2024). However, this process is time consuming. When the protein concentration in the bulk phase was low (10 mg/mL), the diffusion rate was slow, leading to a gradual reduction in interfacial tension (lag regime). After this phase, the gradual adsorption of RG at the interface was observed with a rapid decrease in interfacial tension (rapid decline regime) owing to the minimal electrostatic repulsion and steric hindrance as few protein molecules reached the interface. As more molecules are adsorbed, electrostatic repulsion and spatial hindrance increase, limiting further protein penetration and rearrangement within narrow regions between interfacial proteins (Tao, Cai, Wang, Chen, Zhou, Zhang, & Xu, 2024). The interfacial tension gradually decreased and eventually approached equilibrium (slow-decline regime), whereas this process may take hours or even days (Shen et al., 2024). As shown in Fig. 4A, the interfacial tension at the air-water interface did not reach equilibrium within the 2000-s measurement period, continuing to decrease slowly, while the oil-water interface (Fig. 4B) tended toward equilibrium. This difference might be attributed to the dissimilarity in protein molecule rearrangement rates at the air-water or oil-water interface (Table 2). Previous studies have indicated that slow structural rearrangement of proteins impedes the achievement of equilibrium at the interface (Shen et al., 2024), which in turn explains why the interfacial tension of RG molecules at the oil-water interface is more prone to reach equilibrium.

**Table 2:** Adsorption kinetic parameters of RG at the air/oil-water interface under various shear speeds.

Air-water			
Samples	*K*_*d*_ (mN/m^−1^**·**s^-0.5^)	*K*_*p*_ × 10^3^ (s^−1^)	*K*_*r*_ × 10^3^ (s^−1^)
Control 11,000 rpm	0.0229 ± 0.0006^c^	−1.83 ± 0.02^d^	−3.43 ± 0.10^d^
	0.0273 ± 0.0004^b^	−2.03 ± 0.05^c^	−4.79 ± 0.14^c^
12,000 rpm	0.0331 ± 0.0001^a^	−2.39 ± 0.05^a^	−6.04 ± 0.10^a^
13,000 rpm	0.0275 ± 0.0006^b^	−2.13 ± 0.06^b^	−5.56 ± 0.16^b^
14,000 rpm	0.0281 ± 0.00018^b^	−1.44 ± 0.01^f^	−3.23 ± 0.05^de^
15,000 rpm	0.0271 ± 0.0004^b^	−1.62 ± 0.03^e^	−3.12 ± 0.18^e^

Oil-water			
Control	0.2596 ± 0.0052^d^	−1.42 ± 0.08^f^	−4.48 ± 0.09^d^
11,000 rpm	0.3382 ± 0.0076^c^	−2.59 ± 0.12^c^	−5.02 ± 0.09^c^
12,000 rpm	0.4535 ± 0.0036^a^	−3.68 ± 0.12^a^	−6.26 ± 0.13^a^
13,000 rpm	0.3486 ± 0.0055^b^	−3.37 ± 0.15^b^	−5.49 ± 0.13^b^
14,000 rpm	0.3485 ± 0.0054^b^	−2.40 ± 0.08^d^	−5.37 ± 0.12^b^
15,000 rpm	0.3413 ± 0.0042^bc^	−2.08 ± 0.09^e^	−5.52 ± 0.12^b^

**Fig. 5:**
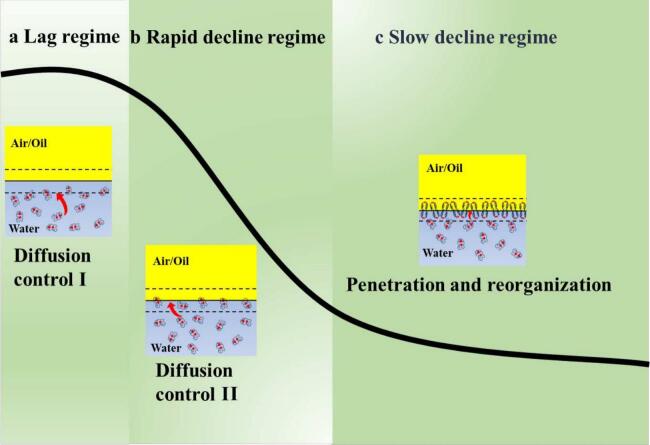
Typical dynamic interfacial tension changes in protein adsorption at the air/oil-water interface over time.

The specific mechanisms underlying these changes are shown in Fig. 5. Although all RG samples exhibited interfacial activity, their conformational changes varied at different shear speeds, resulting in differences in interfacial tension. Furthermore, the compositions of the different interfaces also affect the interfacial tension.

### 3.6 Interfacial adsorption kinetics

Adsorption of protein molecules from the bulk phase to the interface typically occurs in three stages: diffusion, penetration, and rearrangement. Throughout this process, the slopes changed continuously, reflecting the interfacial activity of the molecules and their adsorption kinetics. Table 2 presents the adsorption kinetic parameters of RG at the air/oil-water interfaces under different shear speeds. The shear treatment significantly enhanced the RG adsorption kinetics at both the air-water or oil-water interfaces, increasing the adsorption rate compared with the control. Among the different shear speed groups, the adsorption kinetics at 12,000 rpm were the strongest, indicating that this shear speed most effectively improved the adsorption rate of RG at the interfaces. The diffusion rate (*K*_*d*_), which reflects the initial adsorption capacity of RG at two-phase interfaces, plays a crucial role in the early adsorption behavior (Shi et al., 2024). Compared to the air-water interface, *K*_d_ at the oil-water interface was significantly higher, indicating a faster diffusion rate and stronger initial adsorption capacity at the oil-water interface, consistent with the trend in interfacial tension (Fig. 4). In the control, the *K*_*d*_ values for the air-water or oil-water interfaces were 0.0229 and 0.2596 mN/m^−1^·s^-0.5^, respectively. However, after shear treatment, RG exhibited higher *K*_*d*_ values, peaking at 0.0331 mN/m^−1^·s^-0.5^, for the air-water interface, and 0.4535 mN/m^−1^·s^-0.5^, for the oil-water interface at 12,000 rpm. This suggested that at 12,000 rpm, RG diffused to the interface more quickly, explaining the lower interfacial tension under this shear condition (Fig. 4), because the adsorption activity was closely linked to the diffusion process (Tang & Shen, 2015).

The penetration rate (*K*_p_) and rearrangement rate (*K*_*r*_) of RG at the air-water or oil-water interfaces were assessed. As shown in Table 2, the *K*_*r*_ values for all samples were higher than the *K*_*p*_ values at both interfaces, indicating that conformational rearrangement (*K*_*r*_) played a more significant role than penetration (*K*_*p*_) in the formation of interface films. This pattern is consistent with the observations for other proteins (Tian et al., 2021). Compared to the control (−3.43 s^−1^) at the air-water interface, the *K*_*r*_ of RG increased with shear speed, reaching a maximum absolute value of −6.04 s^−1^ at 12,000 rpm. However, further increases in shear speed caused *K*_*r*_ to decrease below that of the control, likely due to protein re-aggregation, which hindered conformational rearrangement at the interface (Zhu et al., 2024). In contrast, at the oil-water interface, the *K*_*r*_ for all shear-treated RG samples was higher than that of the control, with the maximum absolute value of −6.26 s^−1^ also observed at 12,000 rpm. Since *K*_*r*_ was linked to conformational flexibility and surface hydrophobicity (Fig. 8) and RG exhibited greater conformational flexibility and surface hydrophobicity after treatment (Fig. 2, Fig. 3), these changes promoted the rearrangement of protein molecules at the interface. Additionally, *K*_*r*_ is higher at the oil-water interface than at the air-water interface, likely because the proteins at the oil-water interface can be more prone to undergo conformational changes and rearrange more rapidly (Beverung et al., 1999).

### 3.7 Interfacial expansion rheology

**Fig. 6:**
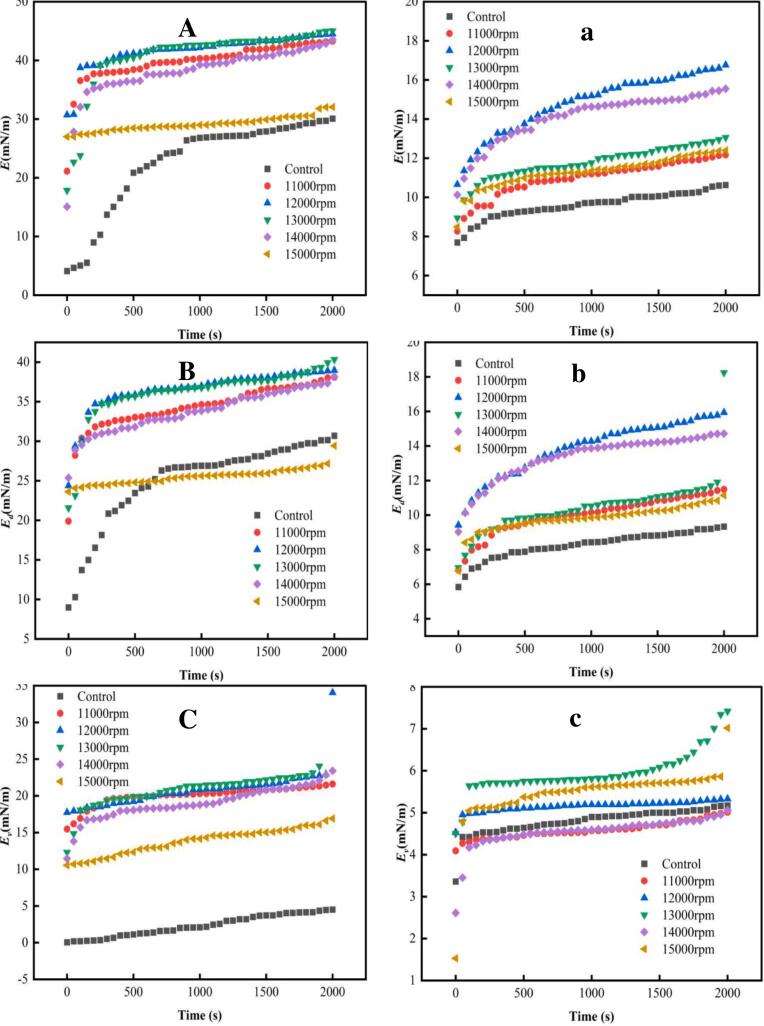
Interfacial expansion rheology of control and HSSH-treated RG at air-water (A) and oil-water (B) interfaces.

Interfacial expansion rheology provides valuable insights into the adsorption behavior of proteins under deformation, shedding light on their role in the stability of emulsions or foams, an aspect that is difficult to assess through interfacial tension alone (Parlak et al., 2024). As shown in Fig. 6, at both the air-water or oil-water interfaces, the interfacial expansion rheology of all RG samples consisted of dilatational modulus (*E*), elastic modulus (*E*_*d*_), and viscous modulus (*E*_*v*_). *E*_*d*_ consistently exceeded *E*_*v*_ and closely approximated *E*, indicating that the two-phase interface films were primarily governed by *E*_*d*_, offering higher resistance to deformation (Sha et al., 2021). The variation in *E*_*d*_ is closely related to the protein-protein interactions and elasticity of the interfacial film (Tao, Cai, Wang, Chen, Zhou, Yang, & Xu, 2024). Because the interface is saturated by diffusion and penetration, protein rearrangement leads to the development of *E*_*d*_ (Hu et al., 2024). In this study, all groups exhibited similar *E*_*d*_ development: an initial rapid increase, signifying rapid protein anchoring at the bare interface, and early interface structure formation, followed by a slower increase, indicating protein rearrangement and maturation of the interface layer over time (Zhang, Zheng, et al., 2025).

Further studies revealed that, compared with the control, the enhanced conformational flexibility of RG after shear treatment facilitated its penetration and unfolding at the interface. This improved protein adsorption and rearrangement efficiency leads to stronger interactions among protein molecules at the interface (Hu et al., 2024). These changes resulted in a higher *E*_*d*_ for RG after shear treatment, suggesting that sheared RG was more likely to adsorb onto the two-phase interface and form an elastic film with greater mechanical strength, thus enhancing the thermodynamic stability of emulsions and foams (Parlak et al., 2024). Among the shear speed groups, 12,000 rpm demonstrated the highest *E*_*d*_ at both the air-water or oil-water interfaces, attributed to the lower interfacial tension (Fig. 4) and higher *K*_*r*_ (Table 2) under this condition. Therefore, shear treatment at 12,000 rpm promoted stronger interactions between RG molecules at the interface, improving the mechanical strength of the interfacial protein membranes. In contrast, the lower *E*_*d*_ in the control could be attributed to excessive intramolecular/intermolecular disulfide bonds and hydrophobic interactions, resulting in a rigid structure with insufficient flexibility, which negatively affected the adsorption process and weakened the interfacial protein film (Tao, Cai, Wang, Chen, Zhou, Zhang, & Xu, 2024; Zhao et al., 2020). This is consistent with the higher interfacial tension (Fig. 4) and lower *K*_*r*_ (Table 2) observed in the control.

### 3.8 Emulsion and foam microstructure、EAI/ESI and FC/FS

**Fig. 7:**
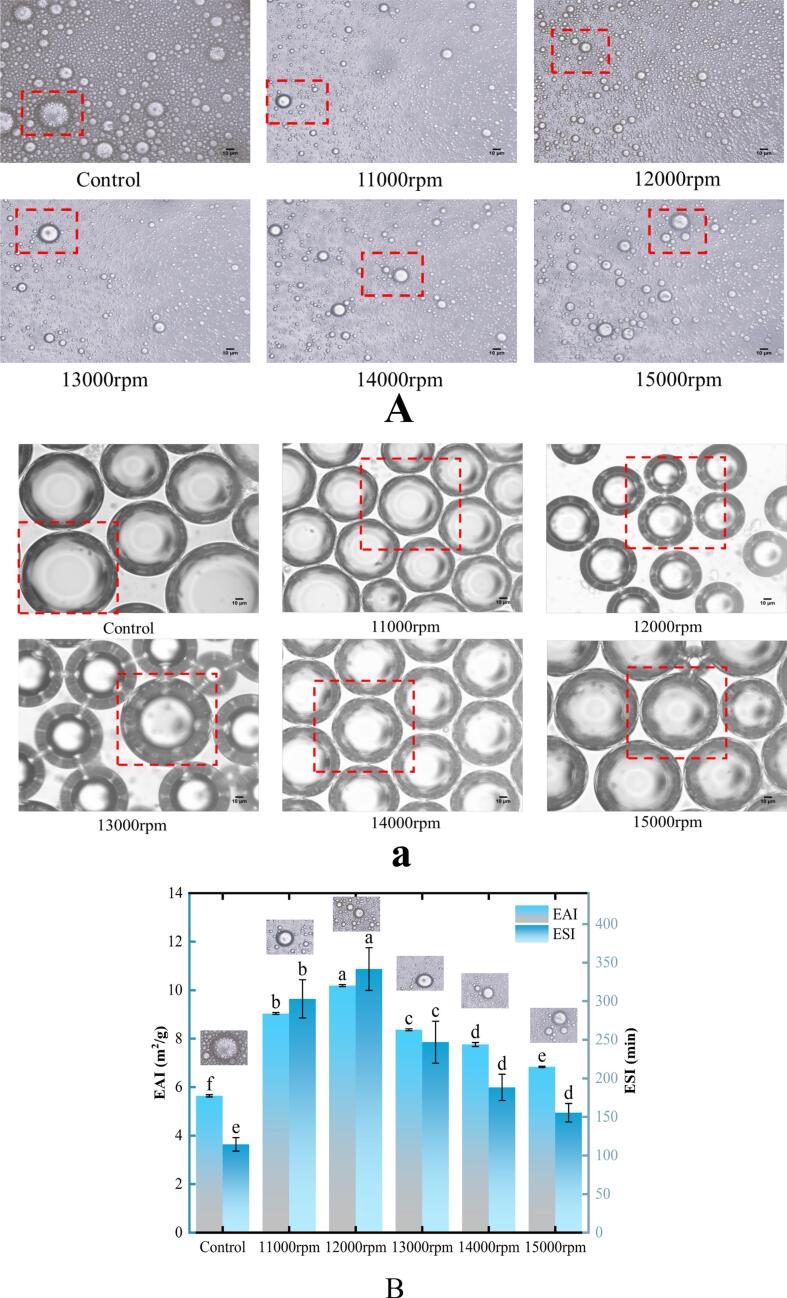

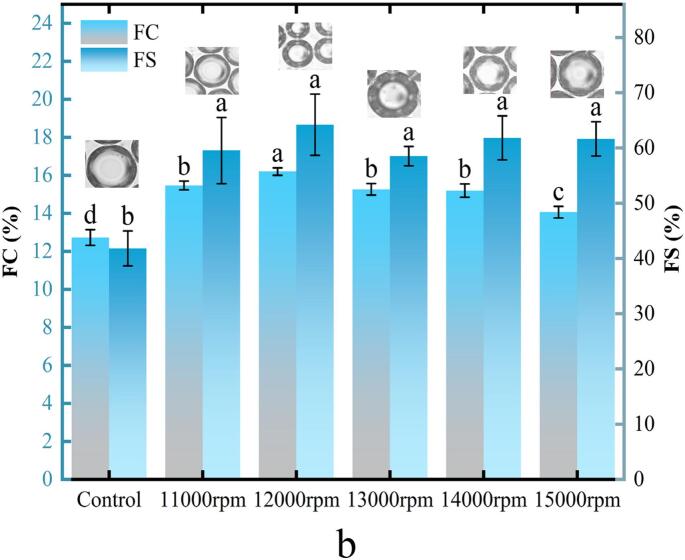
Effect of shear pretreatment at various shear speeds on the emulsion (A) and foam microstructure (a), EAI/ESI (B) and FC/FS (b) of RG.

**Fig. 8:**
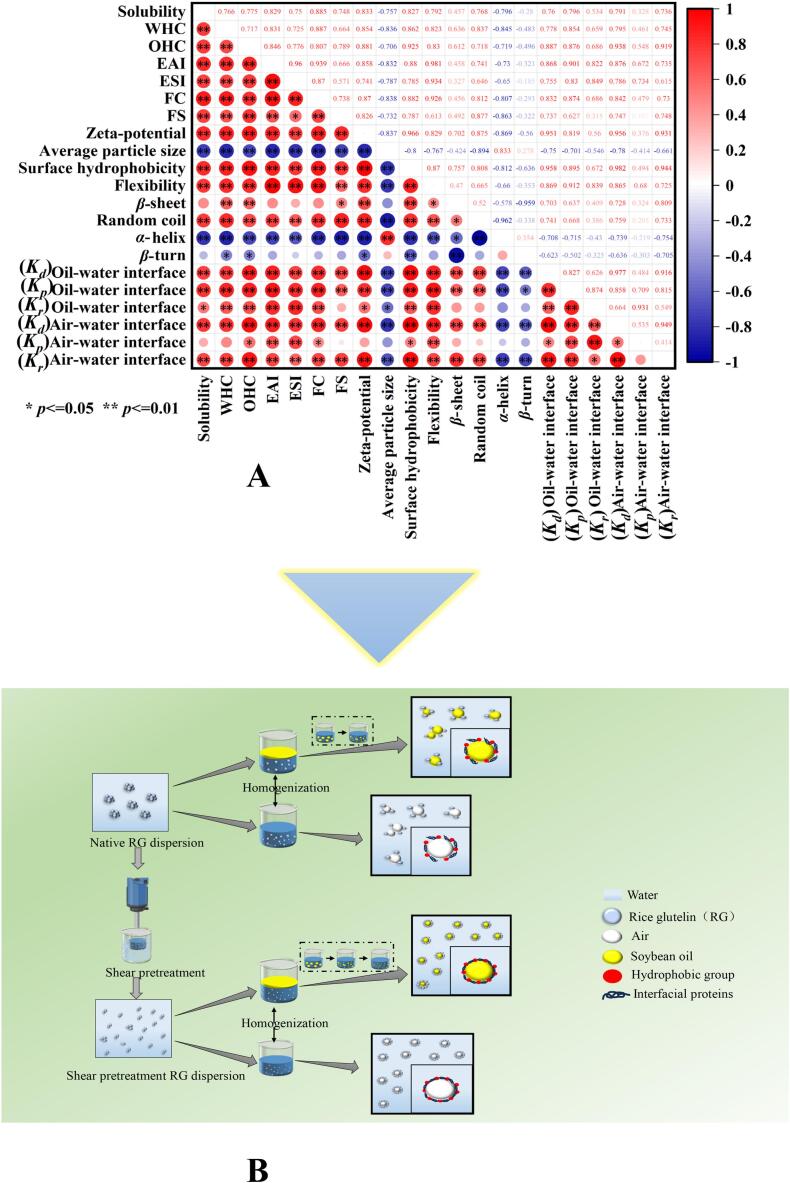
Pearson correlation analysis between different indicators (A); schematic diagram of the changes in the structure, functional properties, and interfacial properties of RG (B).

The sizes of emulsions and foams are generally inversely proportional to their stability. The microscopic structures of the emulsions and foams are shown in Fig. 7A/7a. As the shear speed increased, the sizes of both emulsions and foams decreased, reaching a minimum at 12,000 rpm. Beyond this point, further increases in the shear speed resulted in larger emulsion and foam sizes, indicating that although the shear treatment reduced the size, excessive shear had adverse effects. This trend mirrored the stability of emulsions (Fig. 7B) and foams (Fig. 7b), where ESI (341.98 min) and FS (64.21 %) also peaked at 12,000 rpm, confirming that smaller sizes led to higher stability. This was also associated with a higher *E*_*d*_ (Fig. 6), because stronger interface layers contributed to greater emulsion and foam stability (Niu et al., 2023; Yang et al., 2021). Unlike ESI and FS, EAI and FC were primarily influenced by interfacial tension and adsorption kinetics (Fig. 8A) because these factors play a key role in the formation of emulsions and foams (Martínez-Velasco et al., 2018). After shear treatment, RG exhibited shorter lag times and faster diffusion rates (Fig. 4 and Table 2), leading to greater protein adsorption at the interface, reducing the interfacial tension, and resulting in smaller, more stable emulsions and foams (Fig. 7A and a). Consequently, the EAI and FC values were significantly enhanced, reaching their highest points (10.19 m^2^/g for EAI and 16.20 % for FC) at 12,000 rpm. In contrast, the control demonstrated a poor morphology and low EAI, ESI, FS, and FC values. Thus, faster adsorption and greater protein amounts at the interface led to better elasticity of the interfacial film, and increased stability of emulsions and foams.

### 3.9 Correlation analysis and schematic mechanism

Fig. 8A shows the Pearson correlation analysis of various RG indicators. Zeta potential, surface hydrophobicity, conformational flexibility, and random coil structure exhibited strong positive correlations with solubility (*P* < 0.01), indicating that higher zeta potential, surface hydrophobicity, and conformational flexibility, along with a greater proportion of random coil structures, improved solubility. In contrast, average particle size and *α*-helix structure demonstrated strong negative correlations with solubility (*P* < 0.01), indicating that a larger average particle size and higher proportion of *α*-helix structure, reduced solubility. In addition, the zeta potential, surface hydrophobicity, conformational flexibility, *β*-sheet structure, and random coil structure were highly positively correlated with W/OHC (*P* < 0.01), indicating that higher zeta potential, surface hydrophobicity, and conformational flexibility, along with a greater proportion of *β*-sheet structure and random coil structures, increased W/OHC. In contrast, the average particle size and *α*-helix structure were strongly negatively correlated with W/OHC (*P* < 0.01), and the *β*-turn structure was negatively correlated with W/OHC (*P* < 0.05), indicating that a larger average particle size and higher proportion of *α*-helix and *β*-turn structures, reduced W/OHC. The zeta potential, surface hydrophobicity, conformational flexibility, and random coil structure also displayed strong positive correlations with EAI and ESI (*P* < 0.01), indicating that higher zeta potential, surface hydrophobicity, and conformational flexibility, along with a greater proportion of the random coil structure, enhanced EAI and ESI. In contrast, the average particle size and *α*-helix structure were highly negatively correlated with EAI and ESI (*P* < 0.01), indicating that a larger average particle size and higher proportion of *α*-helix structure, impaired EAI and ESI. Moreover, the zeta potential, surface hydrophobicity, conformational flexibility, and random coil structure were strongly positively correlated with FC and FS (*P* < 0.01), and the *β*-sheet structure was positively correlated with FS (*P* < 0.05), indicating that higher zeta potential, surface hydrophobicity, and conformational flexibility, along with a greater proportion of random coil structures, improved FC and FS, and a higher proportion of *β*-sheet structures enhanced FS. In contrast, the average particle size and *α*-helix structure were strongly negatively correlated with both FC and FS (*P* < 0.01), indicating that a larger average particle size and higher proportion of *α*-helix structures, reduced FC and FS.

EAI and ESI were strongly positively correlated with adsorption kinetics (diffusion, permeability, and rearrangement rates) at the oil-water interface (*P* < 0.01), indicating that stronger adsorption dynamics of protein molecules at this interface lead to improved emulsifying performance, which is consistent with the findings of this study. Similarly, the adsorption kinetics (diffusion and rearrangement rates) at the air-water interface were highly positively correlated with FC and FS (*P* < 0.01), suggesting that faster diffusion and rearrangement of protein molecules at the air-water interface enhanced FC and FS. Interestingly, FC was also positively correlated with the permeability rate at the air-water interface (*P* < 0.05), while the correlation between FS and permeability rate was weaker (*P* > 0.05), implying that the permeability rate played a secondary role in FS compared to the diffusion and rearrangement rates. These results further confirmed that the HSSH treatment significantly altered the functional and interfacial properties of RG by modifying its structure.

Based on the correlation analysis, a schematic diagram was created to illustrate the structural, functional, and interfacial alterations in the RG. As shown in Fig. 8B, the unsheared RG had a larger particle size due to aggregation, which hindered the exposure of negative charges and hydrophilic and hydrophobic groups and limited the conformational flexibility, resulting in weaker interfacial activity at oil-water or air-water interfaces. In contrast, shear treatment promoted protein unfolding, enhanced conformational flexibility, and exposed more hydrophilic and hydrophobic groups and negative charges, which boosted the electrostatic repulsion between protein molecules, reducing the RG size and improving the solubility and water/oil holding capacity. These changes facilitated the faster adsorption of more protein molecules at the interfaces, lowering the interfacial tension and forming a thicker interfacial film, which contributed to the formation of highly stable foams and emulsions.

## 4 Conclusions

This study investigated the impact of HSSH on the structural, functional, and interfacial properties of RG. The results indicated that HSSH treatment, while retaining the primary structure of RG, altered its secondary and tertiary structures, caused partial unfolding of RG, increased surface hydrophobicity, improved conformational flexibility, strengthened electrostatic repulsion, reduced particle size, and formed a loose porous microstructure. These changes led to a remarkable improvement in key attributes, such as solubility, WHC and OHC, EAI and ESI, and FC and FS. Furthermore, the study discovered that the ability of RG to form stable emulsions and foams was closely related to its interfacial characteristics. However, exceeding 12,000 rpm during shear treatment induced protein aggregation, adversely affecting its functionality and interfacial performance.

In conclusion, HSSH pretreatment effectively enhanced the functional and interfacial properties of RG, although further exploration is needed. In particular, the molecular interactions between HSSH and RG remain unclear, and the long-term stability and industrial applications of pretreated RG require further investigation. More comprehensive studies are essential to fully understand and optimize this pretreatment method.

## CRediT authorship contribution statement

**Zhuangpeng Wang:** Writing – review & editing, Writing – original draft, Resources, Formal analysis, Conceptualization. **Zhangtao Chen:** Investigation, Data curation. **Lufan Tan:** Supervision. **Jin Tu:** Validation, Project administration. **Yong Sun:** Software, Resources. **Yuanping Ye:** Resources. **Senwang Zhang:** Validation, Software. **Leiyan Wu:** Writing – review & editing, Funding acquisition.

## Declaration of competing interest

The authors declare that they have no known competing financial interests or personal relationships that could have appeared to influence the work reported in this paper.

## Data Availability

Data will be made available on request.

## Appendix A Supplementary data

Primary constituents of rice protein Image 1
