# Supplementary material for: Impact of high-speed shear homogenization pretreatment on structure, functional characteristics, and interfacial properties: A case of Rice Glutelin
Source: Food Chem X. 2025 Jan 25;25:102219. doi: 10.1016/j.fochx.2025.102219 (PMC11838111; doi:10.1016/j.fochx.2025.102219)
Supplement: Supplementary file 1 — Primary constituents of rice protein [file mmc1.docx]

**Supplementary table 1**

**Table S1**

Primary constituents of rice protein.

| Main composition | Crude protein | Crude fat | Moisture | Ash |
| --- | --- | --- | --- | --- |
| Percentage（%） | 81.61±0.51 | 4.14±0.14 | 4.18±0.12 | 1.18±0.02 |
